# Mind–Body Practices for Mental Health in Higher Education: Breathing, Grounding, and Consistency Are Essential for Stress and Anxiety Management

**DOI:** 10.3390/healthcare13162049

**Published:** 2025-08-19

**Authors:** Kristian Park Frausing, Manja Harsted Flammild, Jesper Dahlgaard

**Affiliations:** 1 Educational Program for Psychomotor Therapy, VIA University College, 8900 Randers, Denmark; mafl@via.dk; 2 Research Program for Mental Health, VIA University College, 8200 Aarhus N, Denmark; jesd@via.dk

**Keywords:** student mental health, mind-body practices, coping strategies, psychomotor therapy, breathing exercises, grounding

## Abstract

**Background and objectives**: Mental health issues such as anxiety and stress are prevalent in educational settings, highlighting the need for individualized, scalable interventions. Mind–body approaches are promising for distress management, and this study explored which body-based strategies students found effective. **Methods**: A cross-sectional study assessed mental health and the use of body-based coping strategies among 152 primarily female students, age 21–52, in the Educational Program for Psychomotor Therapy, a group familiar with such strategies. An electronic survey assessed well-being (WHO-5), stress (PSS-10), anxiety (HADS-A), and use of 13 mind–body practices (e.g., breathing, grounding, muscle relaxation). Participants were grouped by mental health risk and a logistic regression explored associations with coping strategy use. **Results**: High-frequency use of more body-based strategies predicted lower odds of being in the high-risk group (*p* = 0.039), while sporadic use of more strategies predicted high mental health risk (*p* = 0.022). Breathing and grounding were the most frequently used and helpful practices, with minimal barriers. High-risk students cited capability concerns and time as barriers, while all participants mentioned forgetting to use the practices. **Conclusions**: High-risk students use a broader range of practices sporadically, whereas low-risk students adopt selected strategies more consistently. Proper integration of practices through education and training may be crucial for enhancing their efficacy.

## 1. Introduction

### 1.1. Background

Mental well-being among students in higher education has become a key public health concern, with substantial evidence pointing to elevated levels of stress, anxiety, and depressive symptoms in this population [1,2]. In Denmark, where this study is based, recent reports indicate that 29% of first-year students are at risk of poor mental health, and more than 50% of young adults report feeling stressed most of or all the time [3,4], emphasizing the critical need for effective interventions. Elevated distress levels not only have deleterious health effects and reduce quality of life but are also associated with higher educational dropout rates [5].

Mental health interventions have often relied on models of stress and emotion regulation that emphasize cognitive processes, such as Lazarus and Folkman’s [6] transactional model that frames stress as a function of the individual’s cognitive appraisal of environmental demands and perceived coping resources (see Wu et al. [7] for a recent example), or Gross’s [8] process model of emotion regulation that emphasizes deliberate, top-down strategies such as attentional deployment and cognitive reappraisal (for instance, Brady et al. [9]). In turn, psychological counselling has been the most common form of support offered in higher education settings.

However, providing individualized counselling to large student populations is costly and may not be the most effective first line of intervention [1]. Furthermore, while cognitive models have been foundational in shaping mental health research and intervention design, they may offer only a partial account of how distress is regulated in everyday life. A growing body of research highlights the importance of bottom-up, body-mediated mechanisms in the regulation of stress and emotion. In this view, coping is not solely a function of cognitive reinterpretation, but also of sensory awareness, autonomic regulation, and embodied self-experience [10,11].

Newer theoretical frameworks conceptualize self-regulation as a capacity that emerges from the ability to perceive, interpret, and respond to internal bodily signals—such as tension, breath, or heartbeat—through appropriate somatic strategies [11,12]. For instance, the active inference framework offers a promising integrative model in which perception, action, and regulation are seen as continuous processes of aligning expected and actual bodily states [13]. These embodied frameworks provide a rationale for embedding body-based interventions into broader mental health support systems in higher education.

In line with these perspectives, models of mental health promotion drawing on an embodied emotion regulation perspective have been explored in educational contexts, and mind–body practices—such as guided imagery, progressive muscle relaxation, diaphragmatic breathing, and mindfulness-based interventions—have shown promising effects on stress and anxiety reduction [14,15,16,17,18].

Taken together, these developments support a view of coping and emotion regulation that is not exclusively cognitive but deeply embodied, emphasizing the regulatory role of strategies rooted in the body. However, these findings also reveal substantial variability in intervention components, leaving questions about the underlying mechanisms of change and which specific strategies are most effective [16]. While structured mind–body interventions have received some empirical support, little is known about the perceived helpfulness of discrete strategies, or how individuals with training in such methods spontaneously apply them in everyday contexts. In particular, few studies have examined the everyday application and perceived utility of specific mind–body practices outside of formal interventions (though see Winter et al. [19] for an example). This knowledge is essential for designing targeted interventions that are helpful in students’ everyday lives.

### 1.2. The Present Study

Students enrolled in the Educational Program in Psychomotor Therapy represent a population with training in body-based coping strategies. Psychomotor therapy is a mind–body approach that addresses attentional and emotional regulation by promoting present-moment focus and bodily awareness through techniques such as grounding, breathing, visualization, and muscle relaxation [20]. Therapists are seen as role models embodying the skills they teach in clinical settings [21]. Therefore, students are expected not only to acquire theoretical knowledge but to embody these practices through personal experience as a foundational aspect of their professional development. Moreover, given their demographic profile—primarily young women in higher education, a group at elevated risk for mental health issues—these students offer a unique opportunity to investigate the spontaneous, self-directed use of mind–body strategies in a high-risk yet skilled population.

Understanding how psychomotor therapy students apply their training to manage personal stress and anxiety can yield important insights into the utility of embodied coping strategies. It can also help to identify barriers to consistent use, even among individuals with training, thereby informing how such practices might be more effectively taught, integrated, and adapted for broader populations.

### 1.3. Aims and Research Questions

This study explores how students in a psychomotor therapy program use body-based strategies to manage stress and anxiety in everyday life. While structured mind–body interventions have shown promise, little is known about the perceived usefulness of specific strategies when applied independently and outside of formal intervention settings, particularly among individuals trained in such methods. By focusing on this population, the study aims to identify which strategies are perceived as helpful, accessible, and applicable in real-life coping situations.

To address this aim, the study investigates the following research questions:

RQ1: Which body-based strategies do psychomotor therapy students report using most frequently to cope with stress and anxiety in daily life?

RQ2: How do students perceive the benefits and barriers associated with using these strategies?

RQ3: Do students with higher levels of psychological distress differ from those with lower distress in terms of frequency of use, perceived helpfulness, and reported barriers?

This exploration of everyday use of mind–body practices among trained individuals contributes to the growing literature on embodied coping. The findings can inform future mind–body interventions in higher education by identifying strategies with high perceived utility and key conditions for their effective implementation and integration into daily life.

## 2. Methods

### 2.1. Procedures

For this study, we employed a cross-sectional design to examine students’ mental health and use of mind–body coping strategies across semesters in the two psychomotor therapy educational programs in Denmark. Data were collected using an electronic survey developed in the online software platform SurveyXact (https://rambollxact.com/surveyxact, accessed 1 December 2022). The survey included a demographics section where participants reported their gender, age, institutional affiliation, and semester. It further comprised two main sections focused on mental well-being and body-based coping strategies. The well-being section used standardized rating scales, while the coping strategies section featured custom-designed questions to assess the respondents’ use of mind–body practices. Reminder emails were sent five days after the initial invitation to encourage completion, and data collection was completed in May 2023.

#### 2.1.1. Questionnaires

To maximize response rates, we employed brief questionnaires. The WHO-5 Well-Being Index [22] was used to monitor students’ well-being. This five-item self-report inventory assesses feelings of happiness, calmness, activity, rest, and engagement over the previous two weeks, using a six-point Likert scale ranging from 0 (at no time) to 5 (all the time). The total WHO-5 score, ranging from 0 to 100, is calculated by multiplying the sum of the items by four. Scores above 50 suggest normal well-being, scores of 36 to 50 indicate a risk of depression or stress, and scores of 0 to 35 signify a severe risk. The average score in the Danish population is 70 [23].

We measured stress levels with the Danish version of the ten-item Perceived Stress Scale (PSS-10) [24], which includes ten items about perceived stress and control. Items are rated on a five-point scale from 0 (never) to 4 (very often), with scores ranging from 0 to 40; higher scores denote greater stress. The Danish National Health Survey 2021 [25], using a cut-off score of 18 to signify high stress, found that 29 percent of Danes had a high stress score with even higher numbers for younger age groups (16–24: 44 percent; 25–34: 36 percent).

Anxiety levels were assessed using a Danish version of the anxiety subscale (HADS-A) from the Hospital Anxiety and Depression Scale [26]. It contains seven items scored on a scale of 0–3 with a total score range from 0 to 21. A score of 8–10 is the recommended cut-off for a possible presence of anxiety and a score above 10 for a probable presence of anxiety.

The internal consistency of the three psychological scales was assessed using Cronbach’s alpha. In the present sample, the WHO-5 Well-Being Index showed acceptable reliability (α = 0.79), the HADS-A demonstrated good internal consistency (α = 0.81), and the PSS-10 exhibited excellent reliability (α = 0.90). All items within each scale contributed positively to internal consistency, and no item increased reliability if removed. These results indicate that the instruments functioned reliably within the study population.

For the second part of the survey, a questionnaire was developed to assess students’ use of body-based strategies learned through the Educational Program in Psychomotor Therapy. A list of exercises was developed through discussion within the research group, which included two psychomotor therapists affiliated with the educational program, and was subsequently tested on a focus group and refined. Participants were asked to rate their frequency of using 13 different exercises from the psychomotor repertoire, such as breathing techniques, grounding exercises, progressive muscle relaxation, and high-pulse activities. Responses were given on a five-point Likert scale ranging from 1 (never) to 5 (always). To accommodate new students unfamiliar with some exercises, an option to indicate lack of understanding was provided, scored as 1 for analytical purposes. Total scores thus ranged from 13 to 65.

The perceived benefits of each exercise were measured on a four-point scale from 1 (not helpful) to 4 (very helpful). Students were only asked about the helpfulness of exercises they had indicated using. Therefore, an average score of perceived benefits was chosen over a sum score.

Finally, a list of seven items was developed to assess perceived barriers to using psychomotor exercises for managing stress or anxiety. Examples include “I do not know any suitable exercises in this category” and “I do not think that I am skilled enough to use these exercises”. An item for “other barriers” was also included, accompanied by a text field for further elaboration. Participants could also indicate “no barriers.” We used the sum of perceived barriers as our measure for analysis.

#### 2.1.2. Data Analysis

Data were analyzed using R (version 4.1.2), a comprehensive open-source programming language and software environment. Internal consistency of the mental health scales was assessed using Cronbach’s alpha. We employed descriptive statistics to summarize demographics, mental health scores, and coping strategies. Because the rating scales were narrow (1–4 or 1–5) in the coping measures, the use of medians tended to obscure item-level differences, with many exercises sharing the same central value. Means were therefore used to allow for more nuanced comparisons of the most frequently used and beneficial exercises. We also summed the total number of reported barriers for each exercise. Relationships between semester progression and coping measures were examined using Spearman’s rho.

For further analysis, participants were divided into two groups based on cut-off scores on the mental health measures, creating a high-risk group (PSS > 17, HADS-A > 10, or WHO-5 < 36, *n* = 73) and a low-risk group (*n* = 74). Because the key coping variables were based on ordinal, self-reported, and custom-developed measures, and data did not meet normality assumptions, we used non-parametric tests for initial group comparisons. Coping measures were examined using Mann–Whitney U tests, and differences in reported barriers were assessed with Chi-square tests of independence.

To further explore the relationship between students’ use of mind–body coping strategies and their mental health risk status, we conducted a binary logistic regression analysis. As this type of analysis does not require normality of the predictors or the outcome variable, it was appropriate for our dataset. Mental health risk status (high-risk vs. low-risk) served as the outcome variable. The primary predictors were three continuous variables reflecting the number of mind–body exercises each participant never used (rating of 1), used with low-to-moderate frequency (2–3), or used with high frequency (4–5). This pattern-based approach was chosen over a single total-use score, as the latter provided substantially less explanatory power and failed to capture the distinct usage profiles suggested by initial analyses and visual inspection of the data.

Year of study was included as a covariate and potential confounder due to its observed association with strategy use and mental health. Additional potential confounders—gender, age, and institutional affiliation—were tested but excluded from the final model, as they showed no significant association with the outcome and did not improve model fit. Variance inflation factors (VIFs) for all predictors were below 1.5, indicating no problematic multicollinearity despite the interdependence among the three usage variables. Model fit was evaluated using residual deviance and Akaike’s Information Criterion (AIC), and predictor significance was assessed using Wald z-tests.

#### 2.1.3. Ethics

This research project was registered and conducted in compliance with the institutional approval granted to VIA University College by the Danish Data Protection Agency. All participants were informed of the purpose of the study through the invitation email. In accordance with the Declaration of Helsinki [27], informed consent was obtained on the survey’s first page which also restated the purpose of the study. Participation was voluntary, and participants were guaranteed confidentiality and anonymity in any published results. No further approval was required for this type of study by the Danish Research Ethics Committees.

### 2.2. Participants

The survey was distributed to all 316 students enrolled in the two Danish educational programs in psychomotor therapy at VIA University College and University College Copenhagen. Email invitations were sent to students’ institutional email addresses. Email address was then used as an identifier to ensure that only psychomotor students replied and to prevent multiple answers. Class instructors assisted in drawing attention to the study. Inclusion criteria were enrollment in the Educational Program in Psychomotor Therapy and willingness to participate in the study. Exclusion criteria were not actively studying despite being enrolled and not completing any of the survey sections.

Forty-five respondents were excluded due to sick leave, maternity leave, or other absences from active study. Of the remaining 271 students invited to participate, 127 (46.9%) completed the survey fully, and 26 (9.6%) responded partially. One partial response was excluded from the analysis due to lack of data beyond consent. The rest were included in the analyses where possible.

The average age of respondents was 28.7 years, ranging from 21 to 52 years. A total of 2 respondents (1.3%) self-reported as non-binary, 21 respondents (13.8%) were male, and 129 respondents (84.9%) were female, reflecting the general gender ratio of the educational program. Students were distributed across the educational program with 55 students (36.2%) being in their first year of study, 46 (30.3%) being in their second year, and 51 (33.5%) being in the final one and a half years (the last semester mainly comprising the bachelor’s thesis).

## 3. Results

### 3.1. Mental Health

The WHO-5. As shown in Table 1, 149 respondents completed the WHO-5, with a mean score of 56.7. A total of 14 respondents (9.2%) fell below the cut-off score of 35 (indicating severe risk of stress or depression), while 32 respondents (21.1%) had a score between 35 and 50 (moderate risk of stress or depression).

The PSS. A total of 146 respondents completed the PSS, with a mean score of 17. A total of 70 respondents (46.1%) had a score of 18 or higher, indicating high-level stress, and 32 respondents (21.1%) had a score between 13 and 18, thereby falling into the mid-range elevated stress category.

The HADS-A. A total of 146 respondents completed the HADS-A, with a mean score of 8. A total of 32 respondents (21.1%) had a score above the cut-off for probable anxiety, and an additional 45 respondents (29.6%) had a score between eight and ten indicating possible anxiety.

### 3.2. Mind–Body Coping Strategies

Analysis was conducted on 145 responses after excluding 7 respondents who completed only the first part of the survey. Of these, 136 (93.8%) reported using psychomotor strategies for well-being promotion in the past month, and 2 additional respondents had used these methods to alleviate distress in the past, but not within the last month.

A total of 130 respondents (94.2%) indicated that they employed at least one of the body-based strategies “often” or “always.” A correlation analysis revealed a slight tendency towards a progressive increase in the use of these practices with advancement in the study program (Spearman’s rho = 0.25, *p* = 0.002). Similarly, the use of exercises “often” or “always” correlated positively with program progression (rho = 0.26, *p* = 0.001), as did having more practices that provided high gains (rho = 0.16, *p* = 0.046). Despite this association, Mann–Whitney U tests showed no significant differences in the average experienced outcome of the exercises between earlier and later semesters.

The most commonly utilized body-based coping strategies were breathing exercises, grounding exercises, and relaxation exercises. These remained predominant across various levels of well-being, stress, and anxiety, with an exception for the low well-being group on the WHO-5, who favored tactile stimulation and awareness exercises over grounding or relaxation.

Not surprisingly, there was alignment between the most utilized, most beneficial, and least barrier-prone exercises. Breathing and grounding exercises consistently ranked highest in usefulness and ease of engagement, followed by high-pulse exercises for benefit and tactile stimulation for minimal barriers. This is shown in Table 2.

As shown in Table 3, the modal answer to the barrier question was that there were no perceived barriers to using psychomotor exercises which is consistent with the observation that almost all students engaged in these mind–body coping strategies. By far the most frequently experienced barrier was “I do not think about it in the situation” which was mentioned more than twice as often as the second most frequent answer, “It is too troublesome or too time-consuming.”

### 3.3. The High-Risk vs. The Low-Risk Group

Comparative analysis between high-risk and low-risk groups via Mann–Whitney U tests revealed no significant differences in the overall usage of the 13 psychomotor practices or in the perceived outcomes.

**Figure 1:**
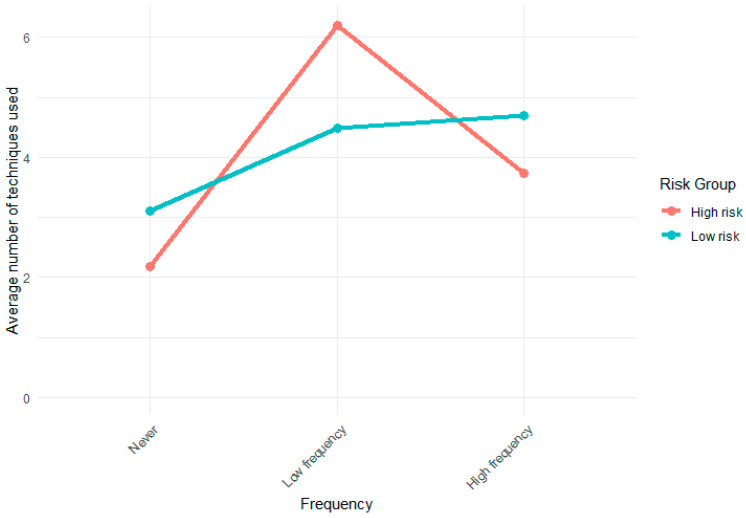
Average number of mind–body exercises never used or used with low or high frequency in the high-risk and low-risk groups.

Initial exploration of pattern-based group differences in coping strategy use showed that students in the low-risk group used more exercises at a high frequency (W = 2196.5, *p* = 0.049), whereas those in the high-risk group used a broader range of exercises at a lower frequency (W = 3664.5, *p* < 0.001). This pattern—more frequent, selective use among low-risk students versus more varied but inconsistent use among high-risk students—is illustrated in Figure 1.

These group-level tendencies were further examined in a logistic regression model (*n* = 142) which also showed that students’ coping strategy usage patterns were significantly associated with their mental health risk group. Specifically, a greater number of moderately used strategies was associated with increased odds of being in the high-risk group (β = 0.161, SE = 0.071, *p* = 0.023), while a higher number of both non-used strategies (β = −0.159, SE = 0.077, *p* = 0.039) and frequently used strategies (β = –0.145, SE = 0.073, *p* = 0.046) were associated with reduced risk. Year of study was not a significant predictor (β = −0.204, SE = 0.229, *p* = 0.373), suggesting that the observed effects of coping patterns were not explained by educational progression. The model achieved a deviance reduction from 203.78 (null model) to 183.74, and an AIC of 195.74, indicating an acceptable fit for exploratory purposes. These findings suggest that students with lower psychological distress were more likely to report using several strategies frequently and others not at all, while students in the high-risk group more often reported moderate or inconsistent use across a broader set of strategies.

Although no significant differences were observed in the total number of perceived barriers between the high-risk and low-risk groups, a detailed comparison revealed distinct patterns. The high-risk group was significantly more likely to report feeling incapable of using the exercises or to describe them as too troublesome or time-consuming. In contrast, the low-risk group was more likely to report contentment with their already adopted practices as a reason for not engaging in additional exercises. The most common barrier across both groups, namely not thinking about the exercises during stressful situations, was reported significantly more frequently by the high-risk group. Conversely, the low-risk group more often reported experiencing no barriers to using the mind–body strategies (Table 3).

## 4. Discussion

Consistent with prior research, this study identified elevated levels of stress and anxiety among undergraduate students, even outside of exam periods, highlighting an ongoing concern about mental health in higher education [28,29,30]. While these challenges have been observed across various disciplines [31,32], the present findings suggest that even students familiar with body-based self-regulation techniques are not immune to such distress. This underscores the need for scalable, embedded mental health strategies within educational settings.

Improving individual mental health literacy is a promising line of intervention [30], and the findings in this study align with previous research supporting the effectiveness of mind–body interventions for stress and anxiety [33,34,35]. Although broader organizational, political, and cultural changes are also essential, the present study focused on the role of individual mind–body coping strategies and their application among students already trained in them. Despite reporting high levels of distress, participants made extensive use of body-based strategies, especially breathing and grounding techniques, which were not only widely applied but also perceived as highly beneficial. These two methods stood out with average helpfulness scores of 3.6 and 3.4, respectively, on a 1–4 scale, and were associated with fewer reported barriers. This is in line with other research indicating the effect of stand-alone mind–body exercises on stress and anxiety [36,37] and suggests that interoceptively oriented strategies, particularly those accessible in the moment without equipment or special settings, may be perceived as effective and applicable tools for everyday distress management.

Breathing exercises have been the subject of previous research, though outcomes may vary depending on how techniques are taught and practiced [37]. Whereas some studies report limited benefit from generic strategies like “taking deep breaths” [33], structured approaches such as diaphragmatic or mindful breathing have consistently shown positive outcomes when implemented as part of stress or anxiety management interventions [37,38], and breathwork has been suggested as one of those accessible and scalable interventions that may ameliorate the present state of global mental health [39].

The psychomotor therapy approach to breathing, which emphasizes observing rather than manipulating the breath, noticing physical sensations associated with it, and thereby increasing awareness of the breath, may enhance interoceptive sensitivity and foster self-regulatory awareness [11]. Within the psychomotor educational program, students are expected to integrate these practices through repeated experiential learning, ensuring both practice and proper instruction—conditions that have been shown to enhance the effectiveness of breathwork interventions [37].

A particularly noteworthy finding was the difference in usage patterns between students classified as high- and low-risk for distress. Students in the low-risk group tended to use selected exercises more consistently, while those in the high-risk group used a wider range of techniques more sporadically. At first glance, this appears counterintuitive, as flexibility in coping has been linked to better psychological outcomes [40]. However, another recent study also found that individuals with higher psychological distress drew on more strategies [41], and our findings suggest that coping flexibility must be accompanied by integration—that is, the internalization of selected practices as habitual, embodied responses. This adds an important nuance to research based on counting the number of strategies as a measure of flexibility [42], supporting the notion that familiarity and embodied engagement with specific techniques may be more critical than the number of techniques known.

From a theoretical standpoint, this distinction resonates with models of interoceptive self-regulation, where effectiveness depends on the individual’s capacity to recognize and respond to internal cues [11,43], and where regulation involves minimizing expected surprise by anticipating and acting to regulate internal and external states [12,13]. From this perspective, coping can become more adaptive when embodied strategies are learned and encoded as prior beliefs within predictive models of bodily states, supporting anticipatory regulation of distress. Students in the high-risk group may not yet have achieved this level of embodied integration, leading to a more reactive and fragmented use of strategies. This interpretation supports other research that shows positive effects of providing young people with opportunities for training and experimenting with coping strategies [44].

The most commonly reported barrier was the failure to remember to use body-based strategies in the moment of distress. This finding was more pronounced among high-risk students, supporting the idea that these techniques must become habitual to be effective when needed most. The low-risk group, who used selected strategies more often, may have developed stronger predictive links between internal distress cues and embodied responses, reducing reliance on conscious recall.

Differences in other reported barriers further underscore this interpretation. High-risk students were more likely to report feeling incapable or overwhelmed about how to apply the techniques, while low-risk students typically described avoiding certain exercises because they were satisfied with the strategies they already employed. This suggests that psychological capability and embodied confidence play a role in whether somatic coping strategies are consistently used. Instructional focus should therefore not only emphasize the availability of strategies but also promote integration, personalization, and mastery over time.

While no between-group differences were found in the perceived effectiveness of the exercises, the different patterns of use and the types of barriers encountered suggest important dynamics in how these strategies function in practice. As such, our findings call for further research into how training, repetition, and self-awareness shape the internalization and utility of mind–body coping methods. Notably, grounding—one of the most frequently used and helpful techniques in this study—remains underrepresented in the empirical literature, despite its centrality in psychomotor and other body-oriented therapies [45,46]. Grounding may offer a uniquely embodied route to emotion regulation that deserves further study in interventions targeting anxiety and stress in educational settings, thereby following recommendations for youth programs to support youth in locating not just external but also internal resources [47].

Finally, our results point toward a broader tension: while students widely use and value body-based strategies, high levels of stress and anxiety persist. This suggests that such techniques may help to regulate symptoms as they arise, but do not necessarily address the structural or environmental causes of distress. Mind–body interventions, therefore, should be seen as part of a multi-level approach, complementing—not replacing—organizational, curricular, and cultural efforts to promote mental well-being in higher education.

### Study Limitations

Some limitations to this study should be acknowledged. First, its correlational design prevents conclusions about causality. While our findings suggest that consistent use of selected body-based strategies is associated with lower distress, it remains unclear whether such coping patterns contribute to improved well-being or simply reflect the greater regulatory capacity of students already experiencing lower levels of distress. Conversely, students in the high-risk group may engage in more diverse but inconsistent coping efforts as a reactive attempt to manage symptoms that feel unresponsive to standard strategies.

Second, although the observed group differences in usage frequency and barriers offer meaningful insights, the homogeneity of the sample limits the scope of interpretation. All participants were students enrolled in a psychomotor therapy program, with advanced knowledge and training in body-based methods. This population likely has greater mental health literacy, self-awareness, and technique familiarity than the general student population. While this focus strengthens the internal validity of the findings, it also raises the possibility of ceiling effects—that is, high baseline use of coping strategies across participants may have masked more subtle differences in usage patterns or perceived effectiveness.

Third, and related to the above, the sampled participants in this study reflect a specific educational and professional context, which may introduce a selection bias and limit the generalizability of the findings. The participants’ familiarity with mind–body strategies and presumed openness to these approaches may make them differ systematically from other student groups in coping behaviors. However, the focus on this population also represents a deliberate strength of the study as the purpose of the study was to explore everyday mind–body strategy use among competent users. Their responses offer important insights into which strategies are most likely to be applied. In a planned intervention study, partly informed by the present study, we will test mind–body coping strategies in a more diverse population.

## 5. Conclusions and Implications

Though caution is warranted regarding generalizability, this study contributes further evidence of mental health challenges in higher education and offers insights into potentially helpful strategies for alleviating student distress. Specific body-based practices, such as breathing and grounding techniques, were perceived as helpful, and the findings suggest that students with better mental health use their repertoire of strategies differently than those experiencing higher distress. Consistent use of a selected, yet varied, set of strategies may be more beneficial than employing all known strategies indiscriminately. One possible interpretation is that proper training and integration are key elements for successful emotion regulation through body-based methods. This interpretation is supported both by the pattern of experienced barriers and by the—admittedly small—increase in frequent strategy use and perceived helpfulness with educational progression, likely reflecting more training and integration over time.

These results offer some implications for intervention development. First, they underscore the practical relevance and accessibility of somatic coping strategies, particularly breathing and grounding techniques. These strategies were not only rated as helpful but also associated with fewer perceived barriers, suggesting they may serve as entry points for developing broader coping repertoires.

Second, the results emphasize the importance of embodied integration over mere strategy familiarity. The observation that students in the low-risk group tended to use fewer strategies more consistently combined with the observation of widespread recall barriers suggests that internalization and routine rather than variety alone may underpin effective self-regulation. Mental health interventions in higher education should therefore include embodied training in programs that emphasize repeated practice and adaptive personalization of strategies, supporting students in moving from occasional use to habitual, embodied deployment. Integrating mind–body exercises into curricula focused on study competences, including stress-management and emotional well-being, may enhance long-term impact on students’ mental health. However, additional research is needed to further explore and substantiate these potential curricular integrations.

At the same time, the study shows that even when body-based strategies are available and used, they do not fully compensate for the sources of distress that characterize student life. This implies that while these practices can enhance self-regulation and momentary coping, they are best understood as complementary tools within a larger ecosystem of support—including relational, institutional, and cultural factors that shape students’ mental well-being.

Finally, the results are directly relevant for the ongoing development of a targeted psychomotor intervention for emerging adults experiencing subclinical anxiety. Such a program should focus not only on the diversity of techniques introduced but also on the depth and consistency of practice, supporting students in gradually building a flexible and embodied coping repertoire. Intervention studies would benefit from including measures of interoceptive awareness, embodied skill acquisition, and habit formation to clarify the mechanisms that make body-based strategies effective and sustainable over time.

## Figures and Tables

**Table 1:** Distribution of mental health scores.

	*n* (Total = 152)	%	Mean (SD)
WHO-5 0–35 36–50 50+ NA	14 32 103 3	9.2 21.1 67.8 2.0	56.7 (15.9)
PSS 0–12 13–17 18+ NA	44 32 70 6	28.9 21.1 46.1 4.0	17.0 (7.04)
HADS-A 0–7 8–10 11+ NA	69 45 32 6	45.4 29.6 21.1 4.0	8.00 (3.73)

Note: WHO-5: The World Health Organization Five Well-Being Index; PSS: 10-item Perceived Stress Scale; HADS-A: Hospital Anxiety and Depression Scale—Anxiety subscale

**Table 2:** Mean use, helpfulness of, and perceived barriers to body-based coping strategies.

Type of Exercise Used	Use M (SD)	Helpfulness M (SD)	Perceived Barriers’ Sum
Breathing	3.7 (1.0)	3.6 (0.6)	72
Grounding	3.4 (1.1)	3.4 (0.7)	73
Relaxation	3.2 (1.2)	3.0 (0.8)	94
Body scan	3.0 (1.2)	3.0 (0.8)	89
High-pulse exercises	2.9 (1.2)	3.2 (0.9)	97
Awareness	2.9 (1.3)	3.1 (0.8)	96
Tactile stimulation	2.8 (1.2)	3.1 (0.8)	78
Basic psychomotor exercises	2.8 (1.1)	2.8 (0.9)	85
Boundary exercises	2.7 (1.3)	3.2 (0.8)	82
Centering	2.7 (1.3)	3.1 (0.8)	98
Visualization	2.5 (1.2)	2.9 (0.9)	104
Increasing muscle tension	2.3 (1.2)	2.8 (0.9)	108
Bodily resistance exercises	1.7 (0.9)	2.8 (0.8)	116

Note: Mean use on a 1–5 scale. Mean helpfulness on a 1–4 scale; sum of barriers reflecting the total number of times a barrier to using the exercise was reported across participants.

**Table 3:** Number of times a perceived barrier was indicated as a hindrance to using mind–body exercises.

Perceived Barrier	Times Mentioned in the High-Risk Group (*N* = 61 Participants)	Times Mentioned in the Low-Risk Group (*N* = 66 Participants)	Chi-Square Test	*p*-Value
Do not know these exercises	76	83	*χ*2 = 0.003 df = 1	*p* = 0.953
Not capable enough	95	59	*χ*2 = 11.5 df = 1	*p* < 0.001 *
Do not believe in effect of these exercises	38	37	*χ*2 = 0.209 df = 1	*p* = 0.648
Too troublesome or time-consuming	106	66	*χ*2 = 12.7 df = 1	*p* < 0.001 *
Do not think about it in the situation	211	185	*χ*2 = 4.37 df = 1	*p* = 0.037 *
Satisfied with other coping strategies	38	83	*χ*2 = 13.4 df = 1	*p* < 0.001 *
Other	65	50	*χ*2 = 3.32 df = 1	*p* = 0.068
No barriers	280	355	*χ*2 = 3.94 df = 1	*p* = 0.047 *

Note: df = degrees of freedom. * indicates statistical significance at the *p* < 0.05 level. Frequencies reflect total mentions of each barrier across exercises. Because each participant could indicate the same barrier for multiple exercises, frequencies exceed the number of participants and percentages are not reported.

## Data Availability

The raw data supporting the conclusions of this article will be made available by the authors on request.

## References

[1] Brown J.S.L. (2018). Student mental health: Some answers and more questions. J. Ment. Health.

[2] Lipson S.K., Zhou S., Abelson S., Heinze J., Jirsa M., Morigney J., Patterson A., Singh M., Eisenberg D. (2022). Trends in college student mental health and help-seeking by race/ethnicity: Findings from the national healthy minds study, 2013–2021. J. Affect. Disord..

[3] Danmarks Evalueringsinstitut (2023). Kortlægning af Trivsel på Universiteterne.

[4] Ungetrivselsrådet (2022). Ungetrivselsanalyse.

[5] Hjorth C.F., Bilgrav L., Frandsen L.S., Overgaard C., Torp-Pedersen C., Nielsen B., Bøggild H. (2016). Mental health and school dropout across educational levels and genders: A 4.8-year follow-up study. BMC Public Health.

[6] Lazarus R.S., Folkman S. (1984). Stress, Appraisal, and Coping.

[7] Wu J.R., Chan F., Iwanaga K., Myers O.M., Ermis-Demirtas H., Bloom Z.D. (2025). The transactional theory of stress and coping as a stress management model for students in Hispanic-serving universities. J. Am. Coll. Health.

[8] Gross J.J. (2015). Emotion Regulation: Current Status and Future Prospects. Psychol. Inq..

[9] Brady S.T., Hard B.M., Gross J.J. (2018). Reappraising test anxiety increases academic performance of first-year college students. J. Educ. Psychol..

[10] Chiesa A., Serretti A., Jakobsen J.C. (2013). Mindfulness: Top–down or bottom–up emotion regulation strategy?. Clin. Psychol. Rev..

[11] Price C.J., Hooven C. (2018). Interoceptive Awareness Skills for Emotion Regulation: Theory and Approach of Mindful Awareness in Body-Oriented Therapy (MABT). Front. Psychol..

[12] Seth A.K., Tsakiris M. (2018). Being a Beast Machine: The Somatic Basis of Selfhood. Trends. Cogn. Sci..

[13] Seth A.K., Friston K.J. (2016). Active interoceptive inference and the emotional brain. Phil. Trans. R. Soc. B.

[14] Conley C.S., Durlak J.A., Kirsch A.C. (2015). A Meta-analysis of Universal Mental Health Prevention Programs for Higher Education Students. Prev. Sci..

[15] Rith-Najarian L.R., Boustani M.M., Chorpita B.F. (2019). A systematic review of prevention programs targeting depression, anxiety, and stress in university students. J. Affect. Disord..

[16] Bamber M.D., Schneider J.K. (2016). Mindfulness-based meditation to decrease stress and anxiety in college students: A narrative synthesis of the research. Educ. Res. Rev..

[17] Dawson A.F., Brown W.W., Anderson J., Datta B., Donald J.N., Hong K., Allan S., Mole T.B., Jones P.B., Galante J. (2020). Mindfulness-Based Interventions for University Students: A Systematic Review and Meta-Analysis of Randomised Controlled Trials. Appl. Psychol. Health Wellbeing.

[18] Worsley J.D., Pennington A., Corcoran R. (2022). Supporting mental health and wellbeing of university and college students: A systematic review of review-level evidence of interventions. PLoS ONE.

[19] Winter E.L., deLeyer-Tiarks J., Bellara A.P., Bray M.A., Schreiber S. (2024). Mind–Body Health in Crisis: A Survey of How Students Cared for Themselves Amidst the COVID-19 Pandemic. COVID.

[20] Flammild M.H., Frausing K.P., Réol L., Wiegaard L. (2024). Forankret og forbundet: Grounding i psykomotorisk praksis. Grounding: Kropslig Forankring i Professionerne.

[21] Ogden P., Fischer J. (2015). Sensorimotor Psychotherapy: Interventions for Trauma and Attachment.

[22] WHO Regional Office for Europe (1998). Wellbeing Measures in Primary Health Care/the DepCare Project: Report on a WHO Meeting.

[23] Topp C.W., Østergaard S.D., Søndergaard S., Bech P. (2015). The WHO-5 Well-Being Index: A Systematic Review of the Literature. Psychother. Psychosom..

[24] Eskildsen A., Dalgaard V.L., Nielsen K.J., Andersen J.H., Zachariae R., Olsen L.R., Jørgensen A., Christiansen D.H. (2015). Cross-cultural adaptation and validation of the Danish consensus version of the 10-item Perceived Stress Scale. Scand. J. Work Environ. Health.

[25] Friis K., Jensen M.M., Pedersen M.H., Lasgaard M., Larsen F.B., Jørgensen S.S., Frandsen K.T., Sørensen J.B. (2022). Hvordan har du det? 2021: Sundhedsprofil for Region og Kommuner. Bind 2. Udviklingen 2010–2013–2017–2021.

[26] Christensen A.V., Dixon J.K., Juel K., Ekholm O., Rasmussen T.B., Borregaard B., Mols R.E., Thrysøe L., Thorup C.B., Berg S.K. (2020). Psychometric properties of the Danish Hospital Anxiety and Depression Scale in patients with cardiac disease: Results from the DenHeart survey. Health Qual. Life Outcomes.

[27] (2025). World Medical Association. World Medical Association Declaration of Helsinki: Ethical Principles for Medical Research Involving Human Participants. JAMA.

[28] Storrie K., Ahern K., Tuckett A. (2010). A systematic review: Students with mental health problems—A growing problem. Int. J. Nurs. Pract..

[29] Djernis D., O’Toole M.S., Fjorback L.O., Svenningsen H., Mehlsen M.Y., Stigsdotter U.K., Dahlgaard J. (2021). A Short Mindfulness Retreat for Students to Reduce Stress and Promote Self-Compassion: Pilot Randomised Controlled Trial Exploring Both an Indoor and a Natural Outdoor Retreat Setting. Healthcare.

[30] Campbell F., Blank L., Cantrell A., Baxter S., Blackmore C., Dixon J., Goyder E. (2022). Factors that influence mental health of university and college students in the UK: A systematic review. BMC Public Health.

[31] Chernomas W.M., Shapiro C. (2013). Stress, Depression, and Anxiety among Undergraduate Nursing Students. Int. J. Nurs. Educ. Scholarsh..

[32] Bork S.J., Mondisa J. (2022). Engineering graduate students’ mental health: A scoping literature review. J. Eng. Educ..

[33] King K.A., Singh M., Bernard A., Merianos A.L., Vidourek R.A. (2012). Employing the Health Belief Model to Examine Stress Management Among College Students. Am. J. Health Stud..

[34] Yusufov M., Nicoloro-SantaBarbara J., Grey N.E., Moyer A., Lobel M. (2019). Meta-analytic evaluation of stress reduction interventions for undergraduate and graduate students. Int. J. Stress Manag..

[35] Cozzolino M., Girelli L., Vivo D.R., Limone P., Celia G. (2020). A mind-body intervention for stress reduction as an adjunct to an information session on stress management in university students. Brain Behav..

[36] Blanck P., Perleth S., Heidenreich T., Kröger P., Ditzen B., Hents H., Mander J. (2018). Effects of mindfulness exercises as stand-alone intervention on symptoms of anxiety and depression: Systematic review and meta-analysis. Behav. Res. Ther..

[37] Bentley T.G.K., D’Andrea-Penna G., Rakic M., Arce N., LaFaille M., Berman R., Cooley K., Sprimont P. (2023). Breathing Practices for Stress and Anxiety Reduction: Conceptual Framework of Implementation Guidelines Based on a Systematic Review of the Published Literature. Brain Sci..

[38] Cho H., Ryu S., Noh J., Lee J. (2016). The Effectiveness of Daily Mindful Breathing Practices on Test Anxiety of Students. PLoS ONE.

[39] Fincham G.W., Strauss C., Montero-Marin J., Cavanagh K. (2023). Effect of breathwork on stress and mental health: A meta-analysis of randomised-controlled trials. Sci. Rep..

[40] Cheng C., Lau H.B., Chan M.S. (2014). Coping flexibility and psychological adjustment to stressful life changes: A meta-analytic review. Psychol. Bull..

[41] Baldwin C.R., Schertz K.E., Orvell A., Costello C., Takahashi S., Moser J.S., Ayduk O., Kross E. (2025). Managing emotions in everyday life: Why a toolbox of strategies matters. Emotion.

[42] Heffer T., Willoughby T. (2017). A count of coping strategies: A longitudinal study investigating an alternative method to understanding coping and adjustment. PLoS ONE.

[43] Mehling W.E., Price C., Daubenmier J.J., Acree M., Bartmess E., Stewart A. (2012). The Multidimensional Assessment of Interoceptive Awareness (MAIA). PLoS ONE.

[44] Lang C., Feldmeth A.K., Brand S., Holsboer-Trachsler E., Pühse U., Gerber M. (2016). Effects of a physical education-based coping training on adolescents’ coping skills, stress perceptions and quality of sleep. Phys. Educ. Sport Pedagog..

[45] Vancampfort D., Brunner E., van Damme T., Stubbs B. (2023). Efficacy of basic body awareness therapy on functional outcomes: A systematic review and meta-analysis of randomized controlled trials. Physiother. Res. Int..

[46] Frausing K.P., Réol L., Wiegaard L. (2024). Grounding: En indføring i begrebet. Grounding: Kropslig Forankring i Professionerne.

[47] Gestsdottir S., Lewin-Bizan S., von Eye A., Lerner J.V., Lerner R.M. (2009). The structure and function of selection, optimization, and compensation in middle adolescence: Theoretical and applied implications. J. Appl. Dev. Psychol..

